# Shall Opticians Attempt to Fit Glasses?

**Published:** 1896-08

**Authors:** 


### SHALL OPTICIANS ATTEMPT TO FIT GLASSES?

Pilgrim, in *The Refractionist, II. No.* 9.

The author says in conclusion: "It is my unflinching belief that there already exists in most of the States sufficient statutory enactments to put an end to the abuses complained of *if only they were vigorously enforced.* Any law which punishes a druggist for counter-prescribing, or the sojourning quack for practicing, ought by any fair construction, to be adequate to reach and punish the refracting optician. At all events, is it not our solemn and bounden duty to test the efficacy of such laws, and, if found inefficient to demand further and more effective legislation? Let the Legislatures of the several States be given clearly and pointedly to understand that such legislation is not invoked for our selfish benefit or protection. Oculists, as a class, are doing well enough now, and do not need the fostering asistance or protection of the law. *But the public need it*, and in its name we should make our demand. And in this effort have we not a right to expect our professional colleagues—the general practitioners—

to stand shoulder to shoulder with us as we in similar movements have stood with them? If, as the guardians of the health and lives of the community, they conceived it to be their duty to restrain, through the mandate of the law, the druggist and the quack from maltreating the public, can they afford to remain indifferent or inactive in a contest which has for its ultimate object the protection and conservation of the sight of their patients?

"And, finally, if the dumb animals and the teeth of the community are worthy of protection against the onslaughts of incompetency; if druggists and quacks are restrained from tampering with other parts of the human body, is it fair to discriminate against one only of its members by withholding from it every semblance of protection? Nay, more, is it not unreasonable as well as unjust to leave the eye, in structure and function perhaps the most delicate organ of the body, a defenseless prey to the assaults of ignorance and the rapacity of human greed."

